# A greenhouse experiment partially supports inferences of ecogeographic isolation from niche models of *Clarkia* sister species

**DOI:** 10.1002/ajb2.1756

**Published:** 2021-10-18

**Authors:** Kaleb A. Goff, Cormac Martinez Del Rio, Kathleen M. Kay

**Affiliations:** ^1^ Department of Ecology and Evolutionary Biology University of California Santa Cruz CA 95060 USA; ^2^ Present address: Kaleb A. Goff, Department of Plant and Microbial Biology North Carolina State University Raleigh NC 27695 USA

**Keywords:** *Clarkia*, ecogeographic isolation, niche divergence, Onagraceae, reciprocal transplant, speciation, species distribution model

## Abstract

**Premise:**

Ecogeographic isolation, or geographic isolation caused by ecological divergence, is thought to be of primary importance in speciation, yet is difficult to demonstrate and quantify. To determine whether distributions are limited by divergent adaptation or historical contingency, the gold standard is to reciprocally transplant species between their geographic ranges. Alternatively, ecogeographic isolation is inferred from species distribution models and niche divergence tests using widely available environmental and occurrence data.

**Methods:**

We tested for ecogeographic isolation between two sister species of California annual wildflowers, *Clarkia concinna* and *C. breweri*, with a hybrid approach. We used niche models to predict water availability as the major axis of ecological divergence and then tested that with a greenhouse experiment. Specifically, we manipulated water availability in field soils for two populations of each species and predicted higher fitness in conditions representing home habitats to those representing the environment of each's sister species.

**Results:**

Water availability and soil representing *C. concinna* generally increased both species' fitness. Thus, water and soil may indeed limit *C. concinna* from colonizing the range of *C. breweri*, but not vice versa. We suggest that the competitive environment and pollinator availability, which are not directly captured with either approach, may be key biotic factors correlated with climate that contribute to unexplained ecogeographic isolation for *C. breweri*.

**Conclusions:**

Ours is a valuable approach to assessing ecogeographic isolation, in that it balances feasibility with model validation, and our results have implications for species distribution modeling efforts geared toward predicting climate change responses.

The importance of ecological divergence across geographic landscapes in driving and maintaining speciation has long been recognized (Turesson, 1922; Clausen et al., 1940; Dobzhansky, 1940; Mayr, 1947; Stebbins, 1950), but is difficult to test. Closely related taxa may be geographically isolated because of historical vicariance or dispersal events, but still share the same fundamental niche. In other words, geographic isolation may be ephemeral with sufficient time and dispersal. Alternatively, when isolated species adapt to local conditions, they may be limited from establishing in their sister species' range because local adaptation may come at the cost of broad tolerance to the conditions within the sister species' range. Thus, even if the species disperse into each other's ranges, they will not establish, and geographic isolation will be maintained. This ecogeographic isolation, or geographic isolation due to divergent ecological adaptation, may function as a common mechanism of prezygotic reproductive isolation (Ramsey et al., 2003; Sobel, 2014), given how frequently sister species exhibit allopatric and/or adjacent geographic ranges (Jordan, 1908; Barraclough and Vogler, 2000; Losos and Glor, 2003).

Ecogeographic isolation is often assumed when geographically isolated taxa inhabit distinct environments. Unequivocal evidence for genetically based ecological niche divergence requires laborious reciprocal transplants (e.g., Clausen et al., 1940; Schluter, 1995; Nosil et al., 2002; Angert and Schemske, 2005; Bush and Clayton, 2006). These experiments may be logistically impossible for many mobile, long‐lived, or rare species, especially when considering geographic scale. In recent years, ecogeographic isolation between closely related taxa has often been inferred with species distribution models (SDMs; e.g., McCormack et al., 2010; Glennon et al., 2012; Anacker and Strauss, 2014; Grossenbacher et al., 2014; Sobel, 2014; Hiller et al., 2019; Vargas et al., 2020), co‐opting a widely adopted tool for predicting suitable geographic ranges from the global change literature (Loarie et al., 2008; Wiens et al., 2009; Peterson et al., 2011). However, divergence in SDMs may reflect differences in realized but not fundamental niches, in which case, ecogeographic isolation may be ephemeral and thus overestimated. In addition, models are often based solely on climate variables and may overlook important biotic interactions and fine‐scale abiotic factors that limit distribution (Wiens et al., 2009), leading to underestimation or mischaracterization of ecogeographic isolation. More work is needed that combines niche models with experimental tests of ecological divergence.

Here we used a combined approach to explore the importance of ecogeographic isolation between sister species of plants with adjacent geographic ranges. *Clarkia concinna* (Fisch. & C.A. Mey.) Greene and *C. breweri* (A. Gray) Greene (Onagraceae) are spring‐flowering annuals endemic to the Mediterranean climate region of California (Figure 1). Prior speciation work on this pair has focused on differences in pollination syndrome (Raguso and Pichersky, 1995; Miller et al., 2014; Kay et al., 2019), but ecogeographic isolation may be an important component of reproductive isolation, given the sharp maritime to interior climate gradient that characterizes their joint ranges. Although they are small annual plants in which estimates of lifetime fitness are feasible, field reciprocal transplants are difficult because of the extremely steep and rocky habitat of *C. breweri*. Instead, we examined ecogeographic isolation by combining species distribution modeling with a targeted greenhouse experiment. We first modeled the species' distributions with bioclimatic and soil variables to ask: (1) Which variables best describe each species' niche, and (2) do these variables differ between the species? Our modeling shows that precipitation is the major axis of differentiation and that *C. concinna* receives more annual and winter precipitation than *C. breweri*. Temperature variability is also important, with more extreme seasonal and daily temperatures characterizing the *C. breweri* range. Soil is moderately important in modeling the distribution of *C. breweri*, but is highly variable in both species. We then test these inferences with a greenhouse experiment in field soils in which we manipulate water availability to approximate the mesic versus xeric conditions and growing season length of each species. We cross the water manipulation with home versus away soils for two populations of each species. Under the hypothesis of ecogeographic isolation generated by the SDMs, we predicted that *C. concinna* would have a marked decrease in fitness in a xeric, short‐season treatment, whereas *C. breweri* would have lower fitness in a mesic, long‐season treatment. We interpret our results with additional data characterizing each species' competitive environment and in light of prior work on differences in the pollinator environment.

**Figure 1:**
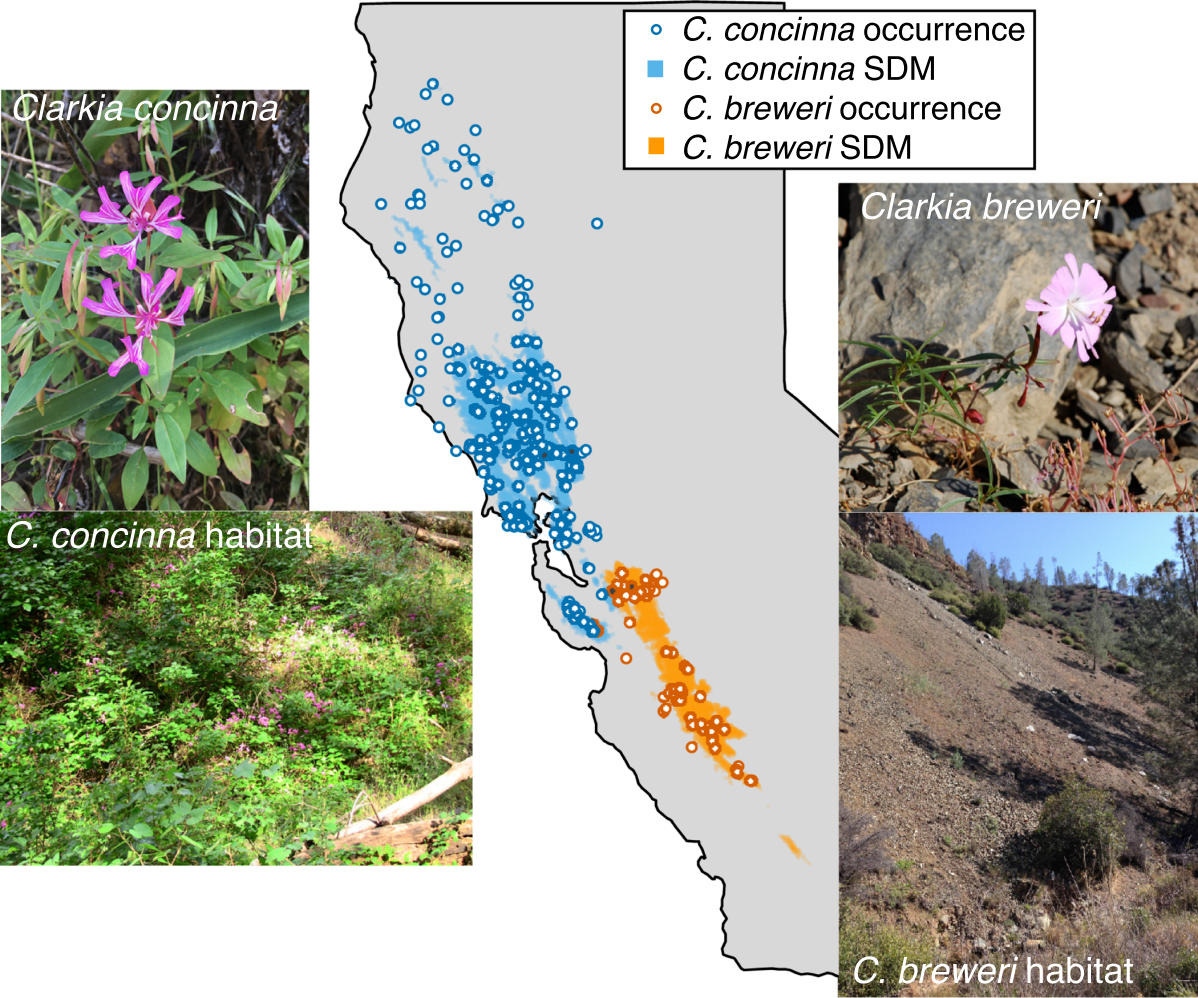
Photographs of *Clarkia concinna, C. breweri*, and their typical habitats, along with a map of occurrences and the 70% probability of occurrence for each species derived from SDMs based on BioClim and soil data. Gray outline shows northern and central California. Sites of seed and soil collection for the greenhouse experiment are represented with gray‐filled circles and were assigned numerical codes from west to east (C1, C2; B1, B2). Photographs by K.M.K. except for *C. concinna* by S. Sianta

## MATERIALS AND METHODS

### Study system

*Clarkia concinna* and *Clarkia breweri* are parapatric sister species endemic to the California Floristic Province (Lewis et al., 1955; Runquist et al., 2016). The ranges of *C. concinna* and *C. breweri* meet on the northeastern slopes of Mt. Hamilton (Santa Clara County, California [CA]). *Clarkia concinna* extends northwest across the coastal range of California for nearly 480 km, where it generally occurs in mesic mixed‐evergreen forest understory. *Clarkia breweri* extends to the southeast for approximately 100 km into San Benito County, CA, where it inhabits steep rocky openings in xeric woodlands and chaparral. Given their range size asymmetry, the occupancy of apparently stressful marginal environments by *C. breweri* and the novelty of its hawkmoth pollination compared to the fly and bee pollination of *C. concinna, C. breweri* has been proposed as a derivative budded species from a *C. concinna*‐like progenitor (Sytsma et al., 1990; Raguso and Pichersky, 1995). Both species germinate in the cool, wet winter and flower and set seed with the onset of the warm, dry summer. Both species are also serpentine tolerators (Safford et al., 2005) occurring across a variety of soils, many of which are notably barren and rocky.

### Species distribution models

We built maximum entropy SDMs for both species with climate and soil data. We downloaded occurrence data from the Global Biodiversity Information Facility (GBIF, http://www.gbif.org) and excluded occurrences without coordinates and those that duplicated collection localities. For four *C. breweri* specimens, we inferred geographic coordinates from precise written descriptions by using online georeferencing software (GeoLocate; https://www.geo-locate.org/). We used a total of 80 specimens for *C. breweri* and 465 for *C. concinna*. The disparity in specimen numbers was expected, since *C. concinna* is much more common and widespread than *C. breweri*.

We used occurrence data for 15 native California vascular plant genera as pseudo‐absence data in the SDMs to represent the range of environmental conditions available to *C. concinna* and *C. breweri*. Using occurrence data that is similarly spatially biased (e.g., observed/collected near roads and on public land) to the occurrence data of the study species produces less‐biased models than when using randomly selected pseudo‐absence data (Phillips et al., 2009). The pseudo‐absence points were bounded by 32° to 45° latitude and –125° to –117° longitude and were subsampled so that there were five pseudo‐absence points per 10‐min resolution grid cell, totaling approximately 1500 points (Appendix S1). Pseudo‐absence points falling east of the California state border were excluded because the Sierra Nevada would be an insurmountable barrier to low‐elevation *C. concinna* and *C. breweri*.

We used WorldClim's 19 bioclimatic variables at 0.5‐min resolution (https://www.worldclim.org/) and soil parent rock types as explanatory variables for the SDMs. The soil raster was derived from a U.S. Geological Survey of California geologic map polygon shape file (Ludington et al., 2005) that we bound within a rectangle in ArcGIS (ESRI, 2011) and rasterized to a resolution of 0.5 min to make it compatible with the BioClim data. The soil raster included both primary, and when available, secondary parent rock types. To build SDMs, we combined the environmental data with the occurrence data for the two species and pseudo‐absence data using the default MaxEnt settings with the package dismo in R (Hijmans et al., 2017). We used 80% of the occurrence data for each *Clarkia* species to train the model and 20% to test its performance. Because many of the BioClim variables are highly correlated (see below), we repeated the SDMs with a subset of less‐correlated BioClim variables, which included isothermality, temperature seasonality, mean temperature of warmest quarter, mean temperature of coldest quarter, annual precipitation, and precipitation of driest quarter. We also repeated the SDMs without the categorical soil data, since the difference in grain size of this data may affect model performance (Manzoor et al., 2018). We assessed and compared models with area under the receiver operating characteristic (ROC) curves (AUC) and by qualitatively comparing the consistency of the percentage contribution of variables to the models.

### Niche comparisons

We compared soil and climate niches separately, since they are fundamentally different types of data. For the soil data, we extracted the primary and secondary parent rock types for each occurrence and compared those constituting at least 10% of occurrences between species with *χ* ^2^ tests. To compare individual climate variables between *C. concinna* and *C. breweri* ranges, we extracted occurrence‐specific values for environmental variables contributing at least 10% to either SDM and compared them with Wilcoxon signed rank tests, since variables were not normally distributed. Although univariate comparisons of BioClim variables seem intuitive, they risk repeated testing of highly correlated variables and do not account for sampling biases in occurrence data or the availability of niche space. Thus, we performed multivariate comparisons of climatic niche by circumscribing each species' occurrence‐based niche relative to available niche space established by pseudo‐absence data using the package ecospat in R (Broennimann et al., 2012). We first extracted values for the 19 BioClim variables from each spatial cell (resolution = 0.5 minutes) included in the geographic extent of the pseudo‐absence occurrence points used to construct SDMs, which represent the available niche space, and conducted a principal component analysis (PCA). We then created a grid with 100 × 100 PCA unit grid cells and used the *Clarkia* species' presence data to project the density of each species into environmental space. This projection uses a kernel density function to adjust the density of occurrences within each cell and reduce the effect of sampling strategy bias when calculating niche differences (Broennimann et al., 2012).

We calculated climatic niche overlap between *C. concinna* and *C. breweri* and tested for niche similarity and niche equivalency (package ecospat; Warren et al., 2008; Broennimann et al., 2012). Niche overlap was calculated as Schoener's *D* (Schoener, 1970). The niche similarity test compares observed niche overlap to a null distribution of simulated overlaps, given the full range of available environmental conditions represented by the pseudo‐absence occurrence points. The niche equivalency test compares observed niche overlap to a null distribution of simulated overlaps when randomly reallocating the occurrences of both species among the joint distribution of occurrences of the two species. Thus, niche similarity indicates whether the two species occupy a similar subset of the total available environment, whereas niche equivalency indicates whether the niches of the two species are statistically distinguishable from each other.

### Greenhouse experiment

Based on the SDMs and niche comparisons, we predicted that water availability is the primary axis of differentiation and that it would be more limiting to *C. concinna*, which experiences a longer and more substantial wet season. Although summer temperatures were important in circumscribing the *C. breweri* niche, *C. breweri* and *C. concinna* set seed before the onset of peak summer temperatures and drought. For annuals with drought and heat escape strategies, water availability likely interacts with temperature, so we chose to manipulate the seasonal availability of water in a gradually warming greenhouse instead of attempting to directly manipulate peak temperatures. Under the hypothesis of ecogeographic isolation generated by the SDMs, we predicted that *C. concinna* would have a marked decrease in fitness in a xeric, short‐season treatment, whereas *C. breweri* would have lower fitness in a mesic, long‐season treatment. Finally, to incorporate the variety of soils on which these species are found and any effects that may have on fitness, we chose to use field‐collected soil in our experiment. Based on our SDMs, we predicted that home soils would have a stronger positive effect on *C. breweri*. We used two populations of each species to evaluate the consistency of water and soil effects within species. Thus, we planted two populations of each species into their home soils and two replicates of their sister species' soil, and we crossed that design with the water manipulation in which we either shortened or extended watering during the growing season. We did not plant each population into the other conspecific population's soil because we were focused on divergent adaptation between species as opposed to local adaptation within species.

We collected seed and soil from two field sites each for *C. concinna* and *C. breweri* (Figure 1). *Clarkia concinna* seed and soil was collected in Chiles Valley, Napa County, CA (C1; WGS 1984: 38.535079 N, 122.33647 W, 261 m a.s.l.) and along a gully crossing Knoxville Road, Napa County, CA (C2; WGS 1984: 38.566285 N, 122.240145 W, 167 m a.s.l.). We collected *C. breweri* seed and soil from Mt. Hamilton, Santa Clara County, CA, about 0.7 km east of the summit (C1; WGS 1984: 37.340047 N, 121.632419 W, 1112 m a.s.l.) and at mile marker 8 along San Antonio Valley Road, Santa Clara County, CA (C2; WGS 1984: 37.3544 N, 121.5601 W, 594 m a.s.l.; Santa Clara County, CA). Fruits from 20 maternal plants were collected from each population. Soil was collected from all field sites by combining shallow samples from the plants' root zone throughout the site and mixing them in bins. Although our soil mixing disrupted soil structure, which may affect water‐holding capacity, we prioritized homogenizing the soil within a treatment.

We added field soil from our four field sites to 120 conetainers (3.8 cm diameter) per site, removing rocks larger than the conetainers. On 15 April 2015, we planted 4–8 seeds from 20 maternal lineages into home‐site soil and the two soil types from the opposing species, and we repeated this process for each watering treatment. We randomized conetainers across racks and germinated seeds in Conviron E‐15 growth chambers (Controlled Environments Limited, Winnipeg, Canada) on a 15°C, 10 h day/10°C, 14 h night schedule where they were hand‐misted daily with deionized water. We censused for germination and survival weekly beginning 05 May 2015. Conetainers were moved to the greenhouse and thinned to the largest plant beginning 21 May 2015. Greenhouse conditions were set to a 25°C day/15°C night schedule, but the greenhouse has limited cooling capacity and exhibited progressively hotter peak daytime temperatures during the experiment, ranging from 25°C to 32°C.

The water treatment was designed to mimic different water availabilities and season lengths and acted as a proxy for the home‐site climate conditions of *C. concinna* (mesic) and *C. breweri* (xeric). We began imposing differences in watering regimes on 28 May 2015, with mesic treatment cones continuing on overhead hand watering every other day until the fourth week, when the cones received water every third day. We continued to water the mesic treatment through 01 August 2015, the 10th week of the experiment. We watered xeric treatment cones every third day the first week, every fourth day the second week, and every fifth day the third week through the sixth week, after which watering ceased.

In the greenhouse, we spaced conetainers on 02 June 2015 to 15 cones per rack. In total, we had 336 conetainers with germinants (70.6% of total planted), representing 204 *C. breweri* and 132 *C. concinna*. Sample sizes by seed source, watering treatment, and soil type are detailed in Appendix S2. We censused flowering weekly between 02 June and 11 August 2015, performing pollinations manually every 2–3 days with a mix of pollen from at least three sires from the same species to ensure differences in seed set would not reflect differences in pollen limitation. Pollen was collected from all available sires of a species with a pipe cleaner, mixed together in a microcentrifuge tube, and then applied to all available stigmas, changing the starting location and direction of our pollination circuit of the greenhouse each time.

We harvested senesced plants from 27 July to 28 August 2015, just before fruit dehiscence. We counted the number of mature seeds and the number of ovules as two measures of fitness and assessed the relationship between these measures to confirm the thoroughness of our pollinations.

We characterized the chemistry and water‐holding capacity of the soils we used in the greenhouse. Samples of the four soils used in this experiment were hand‐sifted to remove the largest rocks, air‐dried and sent to A & L Western Laboratories (Modesto, CA, USA) for analysis of organic matter, estimated nitrogen release, extractable cations, pH, cation exchange capacity, soluble salts and zinc, manganese, iron, copper and boron concentrations. We used conetainers without germinants to test the water‐holding capacity of each soil. To do so, we saturated eight cones from each soil type (C1, C2, B1, B2) with deionized water and measured the wet mass of each cone. Cones were then dried in an oven at 60**°**C for 3 days, weighed again, and the amount of water held was computed. Next, we removed soil from cones and weighed it on the same scale to find the soil dry mass. Finally, we compared wet soil mass and dry soil mass to compute the percentage water‐holding capacity of each soil sample.

We compared fitness of *C. concinna* and *C. breweri* when grown in home soil and water conditions versus the opposing species' soil and water conditions. Ovule number was highly correlated with the number of mature seeds (Appendix S3); thus, a count of the number of mature seeds was used as our measure of lifetime fitness. Conetainers that did not have a germinant were excluded from our analyses, but plants that germinated and did not flower and/or set seed were included, causing significant zero‐inflation in our data set. We analyzed fitness in a full model with species effects and in a model accounting for variation among populations and soil sources without any species effects. For the full model, we used a generalized linear mixed model (GLMM) with a zero‐inflated negative binomial distribution with the package glmmTMB (Brooks et al., 2017). The predictors included plant species, water treatment, soil species, a water treatment by plant species interaction, and a soil species by plant species interaction. The site of soil collection (nested within soil species) and the site of seed collection (nested within plant species) were included as random effects. For the population‐level model, we used a generalized linear model (GLM) with a zero‐inflated negative binomial distribution and a logit link with the package pscl (Jackman, 2008). Predictors included the site of seed collection, the site of soil collection, the water treatment, and the water treatment by seed source interaction. These models allowed us to model the excess zeros contributed by plants that germinated but did not produce seed together with the seed counts of those that did.

### Competitive environment

To explore an additional axis that could help explain niche divergence between species, we compared the competitive environment of *C. concinna* and *C. breweri* habitat using percentage cover data. During the flowering season, we visited four *C. concinna* sites and five *C. breweri* sites (Appendix S4). We concentrated our sampling near the parapatric range boundary to assess habitat‐specific differences rather than differences due to large‐scale climate variation. We placed a 0.25‐m^2^ quadrat around up to 15 plants at least 1 m apart along a linear transect through the center of the population. At two of the five *C. breweri* sites and two of the four *C. concinna* sites, percentage cover by conspecifics, grasses, herbs, leaf litter, or bare ground was visually estimated. At the other sites, a gridded quadrat of 16 points was used, and at each point, a hit was recorded for each ground‐cover type. The number of hits per quadrat was multiplied by 6.25 for each ground‐cover type to calculate percentage cover.

We asked whether the competitive environments for *C. concinna* and *C. breweri* are different. Because none of the percentage cover data was normally distributed, we performed Wilcoxon signed‐rank tests to assess whether the percentage cover differed between the two species' sites for each ground cover type. To visualize variation in the competitive environment across the two species, we used the R package vegan (Oksanen et al., 2020) to do a PCA using the cover types as species data. Finally, we used the vegan package to calculate the Bray–Curtis dissimilarity between all the sites and subsequently test whether the dissimilarity between sites is related to species with a permutational multivariate analysis of variance.

## RESULTS

### Species distribution models

The SDMs built using various combinations of environmental variables all performed similarly well, as indicated by area under the receiver operating characteristic curve (AUC of ROC) scores (Appendix S5). For the SDMs built using all 19 BioClim variables and soil, the AUC of ROC was 0.962 for *C. breweri* and 0.879 for *C. concinna*. The other SDMs created by either dropping highly correlated BioClim variables, soil, or both had slightly lower, but similar AUC values ranging from 0.954 to 0.961 for *C. breweri* and 0.866 to 0.874 for *C. concinna* (Appendix S5). Henceforth, we only discuss the SDMs using all BioClim variables plus soil. The SDMs for *C. concinna* predicted either very low or no probability of environmental suitability for *C. concinna* within the range of *C. breweri* and vice versa (Figure 1; Appendix S6). Moreover, the areas of high predicted suitability very closely overlap the actual species occurrences (Figure 1).

### Niche comparisons

**Figure 2:**
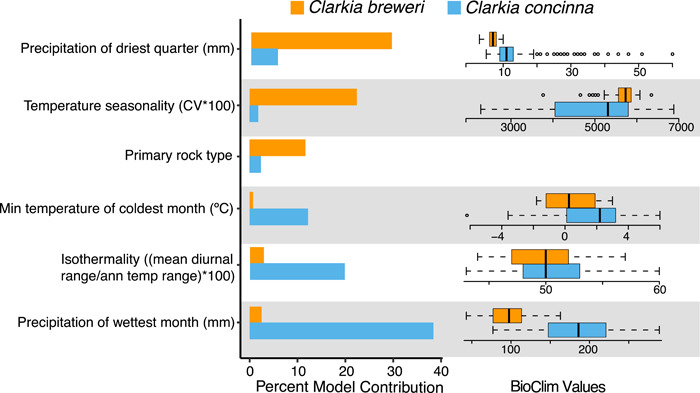
Bioclimatic and soil variables with greater than 10% contribution to either SDM, and boxplots of values for BioClim variables for species occurrences. In univariate comparisons, precipitation of the driest quarter, minimum temperature of the coldest month, and precipitation of the wettest month are significantly higher for *Clarkia concinna*, whereas temperature seasonality is significantly higher for *C. breweri*

**Figure 3:**
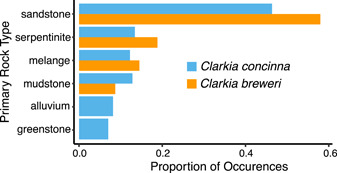
Proportion of occurrences (>0.05) in soils derived from different parent rock types. *Clarkia concinna* and *C. breweri* grow in a variety of soils, and both are found most frequently on sandstone‐ and serpentinite‐derived soils

We first compared variables between species that contributed more than 10% in fitting at least one species' SDM. For *C. breweri*, these variables include precipitation of the driest quarter (29.4%), temperature seasonality (22.4%), and primary rock type (11.7%), whereas for *C. concinna*, these variables include precipitation of the wettest month (38.4%), isothermality (19.8%), and minimum temperature of the coldest month (12.2%; Figure 2). Temperature seasonality is significantly higher where *C. breweri* occurs than where *C. concinna* occurs (Figure 2: Wilcoxon signed rank test: *W* = 9682, *P* < 0.001). In contrast, precipitation of the wettest month, minimum temperature of the coldest month, and precipitation of the driest quarter are higher where *C. concinna* occurs (Figure 2; Wilcoxon signed rank tests: *W* = 29499, *P* < 0.001; *W* = 22550, *P* < 0.001; *W* = 27386, *P* < 0.001, respectively). Isothermality was not significantly different between the two species (Figure 2; Wilcoxon signed rank test: *W* = 16672, *P* = 0.276). The two species each occur in a variety of soils (Figure 3). Although the proportion of occurrences across primary rock types differs by species (*χ* ^2^ = 14.286, df = NA, *P* = 0.0140), the majority of both species are in sandstone‐ and serpentinite‐derived soils.

**Figure 4:**
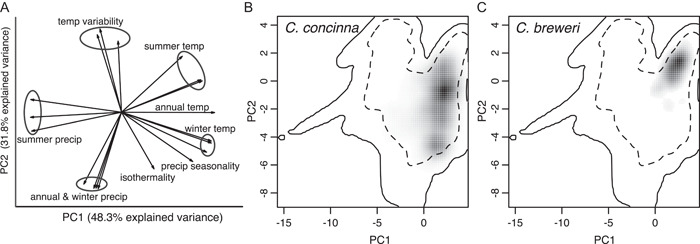
Climate niches of *Clarkia concinna* and *C. breweri*. (A) Biplot from the principal components analysis of the BioClim environmental space defined by psuedo‐absence occurrence data used in the SDMs. Correlated BioClim variables are circled and jointly labeled. (B) and (C) 100 × 100 gridded environmental spaces representing the climate environment available to *C. concinna* and *C. breweri* along these PC axes. Gray shading indicates the density of occurrences within a cell, and dotted and solid lines encompass 50% and 100% of the available environment, respectively. Overall, *C. concinna* experiences more precipitation, less temperature variability, and lower summer temperatures than *C. breweri*. The niches of *C. breweri* and *C. concinna* are significantly different from each other, but they jointly share a similar area of niche space out of the total available environment in cis‐montane California

We then used the PCA of BioClim variables to visualize axes of climate niche differentiation, to project *C. breweri* and *C. concinna* into the available climate niche space, and to perform formal tests of niche differentiation. Within the available climate niche determined by the pseudo‐absence occurrence points, *C. breweri* and *C. concinna* differentiate primarily along PC2, with high values corresponding to high temperature variability and high summer temperatures in *C. breweri* habitat and low values corresponding to high precipitation and mild winter temperatures in *C. concinna* habitat (Figure 4; Appendix S7). *Clarkia concinna* occupies a broader climatic niche space, as expected given its larger geographic range, but there are areas of climate niche space that overlap between the species. The *D* metric of niche overlap for *C. concinna* and *C. breweri* was 0.111, indicating that the two species generally occupy different climates (Figure 4), and this metric was significantly lower than when aggregated occurrences were randomly assigned to species (ecospat test of niche equivalency; *P* = 0.0099; Figure 4; Appendix S8). In contrast, the ecospat test for niche similarity shows that the species' niches are more similar than expected when sampling the available environment at random (*P* = 0.0899). Thus, *C. brewer*i and *C. concinna* occupy significantly different climatic niches in a generally similar area of niche space.

### Greenhouse experiment

**Figure 5:**
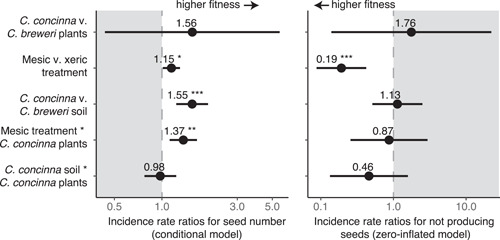
Incidence rate ratios for the effects of species, water treatment, soil source, and their interactions on lifetime fitness (mature seed number) in the greenhouse, with the conditional models of seed number on the left and the zero‐inflated model of the probability of not producing seeds on the right. Error bars represent 95% confidence intervals. Areas of lower fitness are shaded gray. Plants had higher fitness in the mesic treatment and in *C. concinna* soil. The conditional model shows a significant positive interaction term between the mesic treatment and *C. concinna* plants. ^⁎^ *P* < 0.05, ^⁎⁎^ *P* < 0.01, ^⁎⁎⁎^ *P* < 0.001. Full statistical results are in Appendix S9

**Figure 6:**
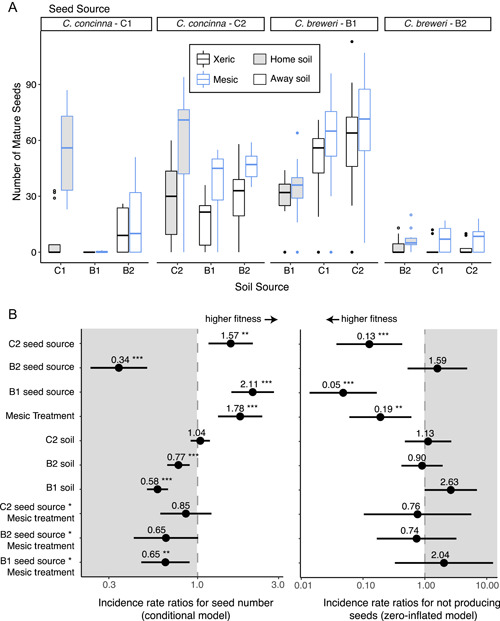
Boxplots (A) and incident rate ratios (B) of seed set by seed and soil site compared to seed set of C1. Populations and soil sources within each species significantly differed in seed set. The mesic water treatment had a positive effect on seed set overall. B1 seed set had a negative interaction with the mesic water treatment. ^⁎^ *P* < 0.05, ^⁎⁎^ *P* < 0.01, ^⁎⁎⁎^ *P* < 0.001. Full statistical results are in Appendix S9

The mesic watering treatment had a positive effect on fitness compared to the xeric treatment in both the conditional and zero‐inflated models and in both the full and population‐level models (Figures 5, 6; Appendix S9). In the full model, the mesic treatment increased the number of seeds by 15% (incidence rate ratio [IRR] = 1.15, *P* = 0.031) and decreased the probability of not producing seeds by 81% (IRR = 0.19, *P* < 0.001) compared to the xeric treatment. In the population‐level model, the mesic treatment increased the number of seeds by 78% (IRR = 1.78, *P* < 0.001) and decreased the probability of not producing seeds by 81% (IRR = 0.19, *P* = 0.005). Nevertheless, the significant positive interaction between the mesic treatment and *C. concinna* (IRR = 1.37, *P* = 0.002; Figure 5) and the significant and marginal negative interactions between the mesic treatment and each *C. breweri* population (B1 × mesic treatment IRR = 0.65, *P* = 0.009; B2 × mesic treatment IRR = 0.65, *P* = 0.054; Figure 6) for seed number show that the fitness benefits of higher water availability are more pronounced for *C. concinna*. Growing in *C. concinna* soil also had a positive effect on seed number in the full model (IRR = 1.55, *P* < 0.001; Figure 5), whereas both *C. breweri* soil sources had a negative effect on seed number in the population level model (B1 soil IRR = 0.58, *P* < 0.001; B2 soil IRR = 0.77, *P* = 0.001; Figure 6). Both species benefitted similarly from *C. concinna* soil, as evidenced by a lack of any species by soil interaction (Figure 5). Finally, we saw pronounced differences in seed set by population in terms of both seed number and the probability of making seeds (Figure 6). One population of each species had relatively high seed set (C2 and B1) compared to the other (C1 and B2), despite conetainers being randomized across the greenhouse. However, the responses of populations within species to water and soil treatments were consistent in direction. Because seed number depends on pollination services in these outcrossing plants, we verified the effectiveness of our hand pollinations by regressing mature seed number on ovule number across all plants. We found a significant positive relationship (*R* ^2^ = 0.9246, df = 334, *P* < 0.001; Appendix S3).

For the four soils we used in the experiment, there were no obviously consistent species‐level differences (Appendix S10). Because we sent a single combined sample from each site for testing, we did not make statistical comparisons among the soils. The soils varied markedly in water‐holding capacity, Ca:Mg ratio, heavy metal concentrations, pH, nitrogen, and organic matter percentage. Most notably, C1 soil had the greatest water‐holding capacity and the lowest Ca:Mg ratio (indicative of serpentine soil), along with moderate heavy metal concentrations and moderate soil nitrogen. C2 had the highest Ca:Mg ratio and a high Cu concentration. *Clarkia breweri* sites were similarly heterogeneous. B1 had the lowest water holding capacity and the second lowest Ca:Mg ratio overall, with high concentrations of Fe and Zn. B2 had the second highest Ca:Mg ratio, the second highest water‐holding capacity, and was the most acidic soil overall.

### Competitive environment

**Figure 7:**
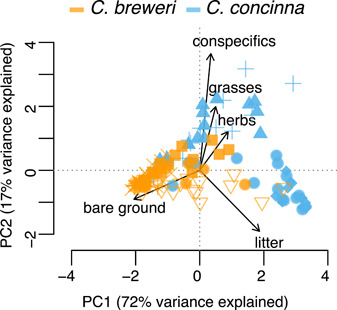
Principal component analysis of ground cover in *Clarkia breweri* and *C. concinna* habitat. Each symbol represents a sampling quadrat, and different symbols indicate different sites (Appendix S4). PERMANOVA shows the species differ in ground cover, and univariate Wilcoxon sign‐rank tests show that *C. breweri* habitat has more bare ground, whereas *C. concinna* habitat has more conspecifics, grasses, herbs, and litter

The competitive environments for *C. concinna* and *C. breweri*, measured by percentage cover of vegetation, litter, and bare ground, differed significantly (Figure 7; PERMANOVA: F. model = 56.852, *R* ^2^ = 0.308, *P* < 0.001). *Clarkia concinna* grows where there are more conspecific, grass, and herbaceous competitors than where *C. breweri* grows (Wilcoxon signed‐rank tests: *W* = 1306.5, *P* < 0.001; *W* = 1200, *P* < 0.001; *W* = 1393.5, *P* = 0.001, respectively). *Clarkia breweri* grows where there is more bare ground (i.e., fewer competitors, *W* = 3835.5, *P* < 0.001). Moreover, in *C. concinna* habitat with few competitors, there is high litter coverage compared to *C. breweri* habitat (*W* = 1251.5, *P* < 0.001).

## DISCUSSION

Ecogeographic isolation is thought to be commonly involved in speciation but is difficult to test and quantify. Whereas allopatry or parapatry are common between sister species (Jordan, 1908; Barraclough and Vogler, 2000; Anacker and Strauss, 2014), it is unclear how often that geographic isolation involves divergent adaptation that would prevent extensive range overlap with enough time and dispersal. Field reciprocal transplant experiments provide the strongest evidence for ecogeographic isolation but are difficult and oftentimes unfeasible. Advances in data availability and computational methods make niche modeling an attractive substitute for documenting ecological divergence among young species (e.g., Grossenbacher et al., 2014; Sobel, 2014; Vargas et al., 2020), but it is unclear how often modeled niches both capture relative axes of niche divergence and represent the limitations of the fundamental niche. With climate change, niche modeling has become indispensable for identifying regions for conservation priority and for predicting range shifts, so it is especially important to understand how modeled niches and ranges relate to fitness differences and, ultimately, population growth rates in nature (Loarie et al., 2008; Wiens et al., 2009; Peterson et al., 2011). In this study, we attempted a hybrid approach in which we modeled the niches of two sister plant species and then experimentally tested the identified major axis of divergence in a greenhouse study. Our results suggest a cautious approach to niche models for inferences of ecogeographic isolation.

For *Clarkia breweri* and *C. concinna*, our SDMs and niche tests show that their niches have diverged in a way that could be responsible for maintaining geographic isolation. *Clarkia concinna* inhabits a more mesic, moderate maritime climate, whereas *C. breweri* occupies more xeric and seasonal interior habitats. Their niches are adjacent in climate space with a small amount of overlap and this is reflected in their parapatric ranges with a narrow band of overlap where their ranges meet.

Do the differences in abiotic factors identified by the SDMs limit the ranges of these species? To answer this question, we chose to focus on water availability as it shows the clearest species‐level difference. Although the range of *C. breweri* is characterized by high temperature seasonality and high summer temperatures, it sets fruit before summer and thus mostly avoids extreme heat. Therefore, the impacts of the temperature seasonality may manifest as a shorter growing season, which we captured in our dry‐down treatments. Although we did not see consistent species‐level differences in soil, considering parent rock or local chemistry and water‐holding capacity at our collection sites, our SDM indicated that soil is moderately important for *C. breweri* and it occurs in steep barren areas with rocky unstable soil. Thus, we used field soils to incorporate some of the edaphic variation in both species and to avoid the unrealistically favorable environment of potting soil.

Overall, our greenhouse experiment shows that *C. concinna* could indeed be limited from expanding into the range of *C. breweri* by water availability and soil conditions, since it experienced fitness declines with the xeric treatment and *C. breweri* soils. Yet our greenhouse results for *C. breweri* were similar to those for *C. concinna* and thus contradict SDM predictions. *Clarkia breweri* had equal or higher fitness with greater water availability and had higher fitness in *C. concinna* soil. Although we saw substantial variation in seed set among populations within both species, which may indicate innate differences in fecundity or variation in mating system and inbreeding depression, the responses to water and soil treatments were remarkably consistent. How can the SDM of *C. breweri* accurately predict its geographic range if the identified abiotic factors are not responsible? It may be that *C. breweri* has established in a drier, more seasonal environment without losing the ability to live in the climate or soil conditions of its putative progenitor species and would not be prevented from colonizing the range of *C. concinna*. Adaptation often proceeds under conditional neutrality, in which adaptation to one environment does not involve fitness trade‐offs to living in another environment (Leimu and Fischer, 2008; Savolainen et al., 2013).

Alternatively, we suggest that the abiotic factors indicate differences in biotic interactions that function as range limiting factors per se. For example, the xeric conditions in the *C. breweri* range may make barren habitat more common, and it appears that *C. breweri* is restricted to relatively bare environments. Therefore, *C. breweri* may be limited from expanding into the range of *C. concinna* by competition in the more heavily vegetated *C. concinna* habitats. In fact, we see this difference in the only sympatric site we have found, where *C. breweri* occupies the steep rocky south‐facing slope of a ravine and *C. concinna* occupies the opposing north‐facing forested slope (Figure 7; filled circles). The apparently benign environment of *C. concinna* may actually represent a more stressful competitive environment for *C. breweri*, although this idea needs to be tested empirically. Trade‐offs between drought tolerance and competitive ability have been found in other plants (e.g., Gurevitch, 1986; Aronson et al., 1993; Liancourt and Tielbörger, 2009) and are similar to the phenomenon noted in edaphic endemics, in which tolerance of stressful edaphic conditions comes at the expense of competitive ability in benign environments (Kruckeberg, 1951, 1967; Cacho and Strauss, 2014; Rajakaruna, 2018).

Modeled climate niche differences may also be a proxy for differences in the pollination environment. *Clarkia concinna* is primarily pollinated by diurnal flies, bees, and butterflies, whereas *C. breweri* is primarily pollinated by nocturnal hawkmoths (Raguso and Pichersky, 1995; Groom, 1998; Miller et al., 2014). Hawkmoth pollination is evolutionarily derived in *C. breweri* and involves a suite of floral traits unique in the *Clarkia* genus (Raguso and Pichersky, 1995). Nevertheless, in a translocation experiment, *C. breweri* was just as attractive as *C. concinna* to diurnal insect pollinators, and the very low rates of diurnal insect visitation to *C. breweri* are due to lower availability of these pollinators in the *C. breweri* habitat (Miller et al., 2014). The most southern populations of *C. concinna* are also highly self‐fertilizing (Bowman, 1987), further suggesting that *C. concinna* is limited from expanding to the south by a dearth of appropriate pollinators. Thus, the divergence in abiotic habitat might underlie divergence in the pollination environment in a way that synergistically increases ecogeographic isolation for *C. concinna*. Moreover, the hypothesized climate‐mediated divergence in pollination environment may have driven the adoption of novel hawkmoth pollinators by *C. breweri*, and this divergence in pollination systems results in strong floral reproductive isolation where the species' ranges meet (Kay et al., 2019), although these species remain interfertile in hand pollinations (Lewis et al., 1955; Raguso and Pichersky, 1995). This type of process has long been hypothesized to be important in plant speciation (Grant and Grant, 1965; Stebbins, 1970) but is difficult to tease apart because it requires understanding the abiotic niche, the pollination niche, and their interactions (Niet et al., 2006; Van der Niet et al., 2014; Phillips et al., 2020).

Our results also support the hypothesis of *C. breweri* being a derivative species from a *C. concinna*‐like progenitor. Progenitor‐derivative, or budding, speciation involves an initially small colonizing population becoming reproductively isolated from a larger‐ranged species, typically in a marginal environment at the range edge (Mayr, 1954; Grant, 1981). Budding speciation with niche divergence is increasingly recognized as important in plants, especially in species‐rich floras like that of the California Floristic Province (Richerson and Lum, 1980; Gottlieb, 2004; Crawford, 2010; Anacker and Strauss, 2014; Grossenbacher et al., 2014). If *C. breweri* is derived from a *C. concinna*‐like progenitor, asymmetric results like ours may be expected, with *C. breweri* evolving to tolerate a novel xeric environment without losing tolerance of a more ancestral environment. However, genetic data are necessary to confirm budding speciation for *C. breweri* (Crawford, 2010).

## CONCLUSIONS

We used niche models to describe and predict ecogeographic isolation between sister plant species and test these models with a greenhouse experiment. Although our models predicted the actual species ranges well, our empirical results were mixed. We found experimental support for the predicted abiotic niche limiting the range of *C. concinna* but not *C. breweri*. Aspects of the biotic environment, including levels of competition and pollinator availability, may be correlated with the abiotic environment but not directly captured by the niche modeling approach. Our results show the difficulty of determining causative factors underlying geographic distributions from niche models alone, although this approach has been widely adopted. Nevertheless, ecogeographic isolation plays an important role in speciation for this species pair and for many others, and it warrants more investigation despite its complexities.

## AUTHOR CONTRIBUTIONS

C.M.D.R. and K.M.K. conducted the species distribution and niche modeling. K.A.G. and K.M.K. conducted the greenhouse experiment. K.A.G., K.M.K., and C.M.D.R. wrote the manuscript.

### Open Research Badge

This article has earned an Open Data badge for making publicly available the digitally‐shareable data necessary to reproduce the reported results. The data are available at https://doi.org/10.5061/dryad.tb2rbp00h (Goff et al., 2021). Learn more about the Open Practices badges from the Center for Open Science: https://osf.io/tvyxz/wiki.

## Supporting information


**Appendix S1**. Pseudo‐absence data points and genera used to generate SDMs and niche comparisons.

**Appendix S2**. Greenhouse experiment sample sizes by species, watering treatment, and soil source.

**Appendix S3**. Regression of ovule number and number of mature seeds.

**Appendix S4**. Table of sites used to characterize the competitive environment.

**Appendix S5**. Tables of SDM AUC scores and complete model outputs using different combinations of BioClim and soil data.

**Appendix S6**. Maps of predictive surfaces generated by SDMs.

**Appendix S7**. Table of BioClim variables and PCA axis loadings.

**Appendix S8**. Niche similarity and equivalency test graphs.

**Appendix S9**. Results tables from models used to analyze greenhouse experiment.

**Appendix S10**. Soil chemistry and water‐holding capacity table.

## Data Availability

All data and code used in this paper are available at the Dryad Digital Repository: https://doi.org/10.5061/dryad.tb2rbp00h (Goff et al., 2021).
