# Supplementary material for: A greenhouse experiment partially supports inferences of ecogeographic isolation from niche models of Clarkia sister species
Source: Am J Bot. 2021 Oct 18;108(10):2002–14. doi: 10.1002/ajb2.1756 (PMC9298282; doi:10.1002/ajb2.1756)
Supplement: Supplementary file 1 — Appendix S1. Pseudo‐absence data points and genera used to generate SDMs and niche comparisons. [file AJB2-108-2002-s004.docx]

**Appendix S1:** Pseudo-absence data used to generate SDMs and for niche comparisons, including a list of the 15 genera used to generate the targeted background pseudo-absence data, and a map of their occurrences within the geographic bounds we specified.

**
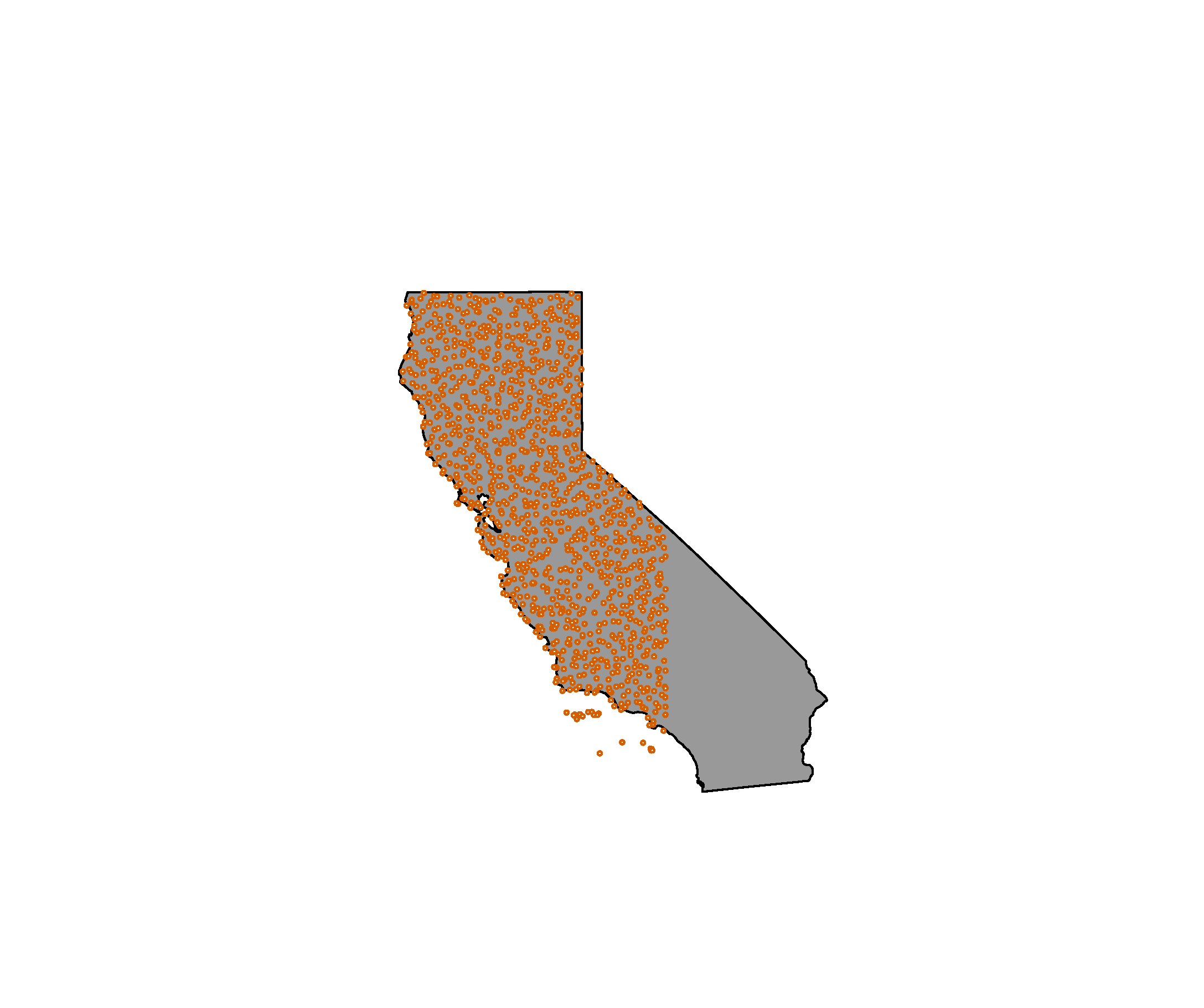
Genus**

*Quercus*

*Lasthenia*

*Elymus*

*Baccharis*

*Yucca*

*Eschscholzia*

*Clarkia*

*Pseudotsuga*

*Brassica*

*Pinus*

*Populus*

*Lupinus*

*Poa*

*Arctostaphylos*

*Pteridium*
