# Supplementary material for: A greenhouse experiment partially supports inferences of ecogeographic isolation from niche models of Clarkia sister species
Source: Am J Bot. 2021 Oct 18;108(10):2002–14. doi: 10.1002/ajb2.1756 (PMC9298282; doi:10.1002/ajb2.1756)
Supplement: Supplementary file 2 — Appendix S2. Greenhouse experiment sample sizes by species, watering treatment, and soil source. [file AJB2-108-2002-s006.docx]

**Appendix S2:** Greenhouse experiment sample sizes by species, watering treatment, and soil source. Seedlings refers to the number of seedlings that we followed for lifetime fitness. Because multiple seeds were planted per conetainer, it does not indicate the overall germination rate.

| Seed Source | Soil Source | Water Treatment | Seedlings |
| --- | --- | --- | --- |
| C1 | C1 | Xeric | 13 |
|  |  | Mesic | 14 |
|  | B2 | Xeric | 8 |
|  |  | Mesic | 15 |
|  | B1 | Xeric | 4 |
|  |  | Mesic | 3 |
| C2 | C2 | Xeric | 11 |
|  |  | Mesic | 11 |
|  | B2 | Xeric | 15 |
|  |  | Mesic | 15 |
|  | B1 | Xeric | 10 |
|  |  | Mesic | 13 |
| B2 | C1 | Xeric | 17 |
|  |  | Mesic | 18 |
|  | C2 | Xeric | 13 |
|  |  | Mesic | 14 |
|  | B2 | Xeric | 15 |
|  |  | Mesic | 14 |
| B1 | C1 | Xeric | 20 |
|  |  | Mesic | 19 |
|  | C2 | Xeric | 19 |
|  |  | Mesic | 18 |
|  | B1 | Xeric | 18 |
|  |  | Mesic | 19 |
