# Supplementary material for: A greenhouse experiment partially supports inferences of ecogeographic isolation from niche models of Clarkia sister species
Source: Am J Bot. 2021 Oct 18;108(10):2002–14. doi: 10.1002/ajb2.1756 (PMC9298282; doi:10.1002/ajb2.1756)

**Appendix S3**: Graph of ovule number and mature seed number, including statistical analysis using linear regression (R^2^ = 0.9246, df = 334, p = <0.001). This result confirms that our hand-pollinations in the greenhouse were successful at transferring enough pollen to the stigma of mature flowers to facilitate fertilization of ovules and development of mature seeds.


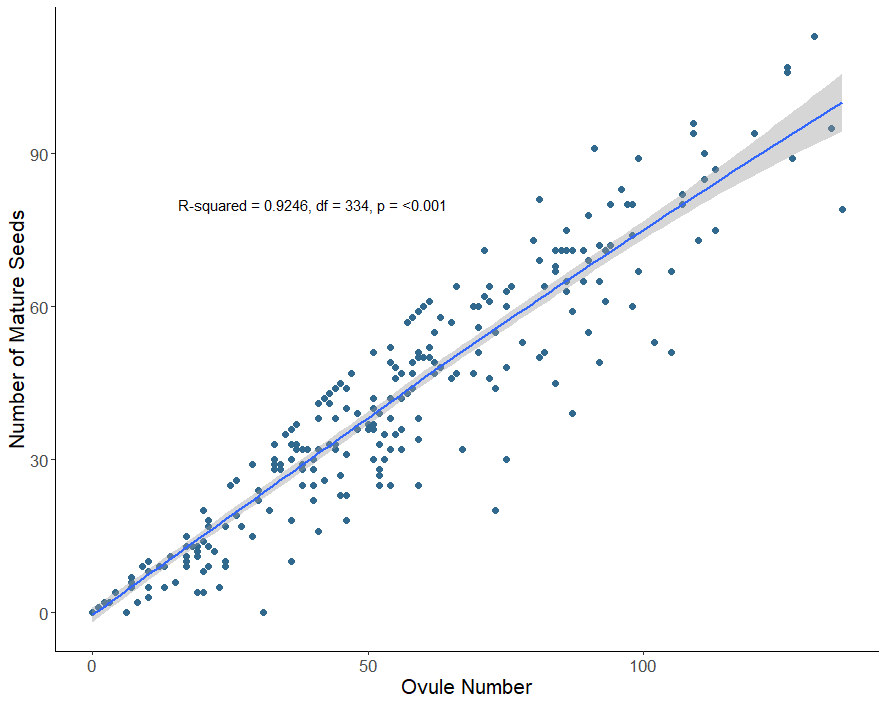

Supplement: Supplementary file 3 — Appendix S3. Regression of ovule number and number of mature seeds. [file AJB2-108-2002-s001.docx]
