# Supplementary material for: A greenhouse experiment partially supports inferences of ecogeographic isolation from niche models of Clarkia sister species
Source: Am J Bot. 2021 Oct 18;108(10):2002–14. doi: 10.1002/ajb2.1756 (PMC9298282; doi:10.1002/ajb2.1756)

**Appendix S4:** Sites sampled to characterize the competitive environment, with a map showing their distribution.

| **Site** | **Latitude** | **Longitude** | **Description** | **Symbol in Fig. 7** |
| --- | --- | --- | --- | --- |
| *C. breweri* |  |  |  |  |
| Mt. Hamilton | 37.3497 | -121.6081 | 3.1 miles east on San Antonio Rd from the summit of Mt. Hamilton | ⚫ |
| San Antonio Valley Rd | 37.3544 | -121.5601 | Mile Marker 8 on San Antonio Rd | X |
| Frank Raines | 37.4198 | -121.3582 | 2 km east of Frank Raines Regional Park | ⃞ |
| Del Puerto Canyon | 37.4134 | -121.4368 | 2 km west of Frank Raines Regional Park | ▽ |
| Pinnacles National Park | 36.4828 | -121.1668 | Bear Gulch Canyon | ✳ |
| *C. concinna* |  |  |  |  |
| Mt. Hamilton | 37.3497 | -121.6081 | 3.1 miles east on San Antonio Rd from the summit of Mt. Hamilton | ⚫ |
| Devil’s Head - Knoxville Rd | 38.8205 | -122.3532 | McLaughlin Natural Reserve | △ |
| Reiff Rd | 38.8722 | -122.4055 | McLaughlin Natural Reserve | ＋ |
| Trumpler’s Garden | 37.3526 | -121.6318 | 0.68 km north of Copernicus Peak on Mt. Hamilton | ♢ |


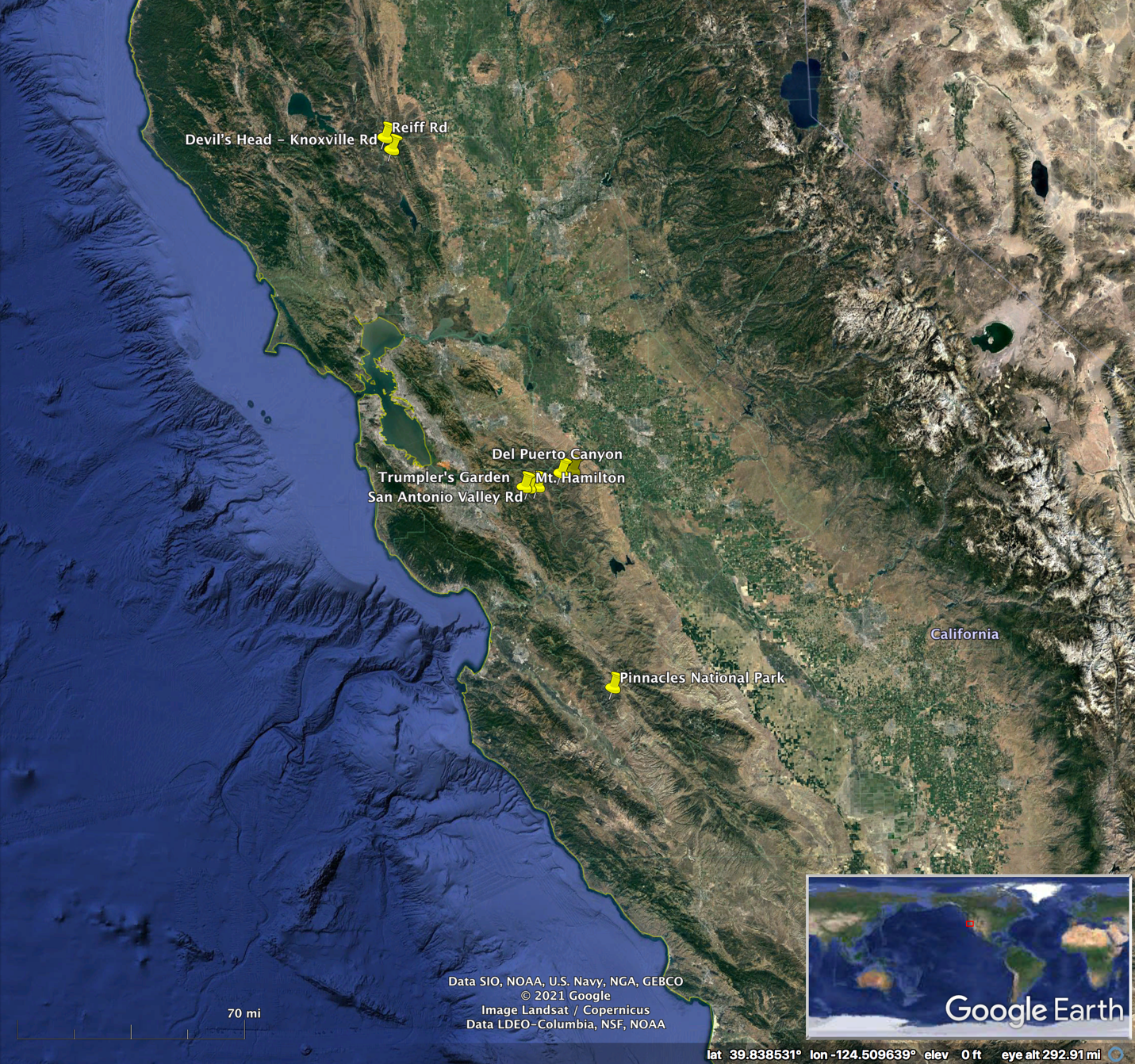

Supplement: Supplementary file 4 — Appendix S4. Table of sites used to characterize the competitive environment. [file AJB2-108-2002-s008.docx]
