# Supplementary material for: A greenhouse experiment partially supports inferences of ecogeographic isolation from niche models of Clarkia sister species
Source: Am J Bot. 2021 Oct 18;108(10):2002–14. doi: 10.1002/ajb2.1756 (PMC9298282; doi:10.1002/ajb2.1756)
Supplement: Supplementary file 5 — Appendix S5. Tables of SDM AUC scores and complete model outputs using different combinations of BioClim and soil data. [file AJB2-108-2002-s005.docx]

**Appendix S5 Table 1**: Area under the receiver operating characteristic curve (AUC of ROC) values created by the test data when building SDMs for both species. “Subset of Bioclim” indicates that only a subset of BioClim variables were used to build the model. The subset included representative variables from the correlate groups of the study area (Temperature Seasonality, Isothermality, Precipitation of the Driest Quarter, Annual Precipitation, Mean Temperature of Warmest Quarter, and Mean Temperature of Coldest Quarter). “Soil” indicates that both primary parent rock type and secondary parent rock type were used to train the model.

|  | All 19 BioClim and Soil | Subset of Bioclim and Soil | Subset of BioClim | All 19 BioClim |
| --- | --- | --- | --- | --- |
| *C. breweri* | 0.962 | 0.958 | 0.954 | 0.961 |
| *C. concinna* | 0.879 | 0.872 | 0.866 | 0.874 |

**Appendix S5 Table 2**: Percent contribution and percent permutation of each environmental variable used to build the four different *C. breweri* MaxEnt models.  “Subset of Bioclim” indicates that only a subset of BioClim variables were used to build the model. The subset included representative variables from the correlate groups of the study area (Temperature Seasonality, Isothermality, Precipitation of the Driest Quarter, Annual Precipitation, Mean Temperature of Warmest Quarter, and Mean Temperature of Coldest Quarter). “Soil” indicates that both primary parent rock type and secondary parent rock type were used to train the model.

|  |  | Model – *Clarkia breweri* | | | | | | | |
| --- | --- | --- | --- | --- | --- | --- | --- | --- | --- |
|  |  | All Bioclim + Soil | | Subset of  Bioclim + Soil | | Subset of  Bioclim | | All Bioclim | |
| Bioclim Variable Correlate Group | Bioclim Variable | % Contrib | % Permut | % Contrib | % Permut | % Contrib | % Permut | % Contrib | % Permut |
| Measures of Temperature and precipitation seasonality | Temperature Seasonality | 22.4 | 3.9 | 32.4 | 12 | 44.1 | 10.5 | 25.4 | 3.3 |
|  | Temperature Annual Range | 0 | 0 | NA | NA | NA | NA | 0 | 0 |
|  | Mean Diurnal Range | 7.2 | 0 | NA | NA | NA | NA | 6.6 | 0 |
|  | Isothermality | 2.9 | 2.9 | 9.2 | 7.5 | 10.5 | 9.9 | 3.8 | 1.9 |
|  | Precipitation Seasonality | 7.9 | 5.2 | NA | NA | NA | NA | 8.5 | 2.9 |
| Measures of precipitation during the driest/warmest parts of the year | Precipitation of Driest Month | 8.3 | 0.1 | NA | NA | NA | NA | 11.2 | 0.2 |
|  | Precipitation of Driest Quarter | 29.4 | 62.8 | 35.4 | 42.5 | 33.8 | 46.4 | 32 | 37.5 |
|  | Precipitation of Warmest Quarter | 0.3 | 0 | NA | NA | NA | NA | 3.1 | 0 |
| Measures of precipitation during the coldest/wettest parts of the year | Annual Precipitation | 0 | 0 | 3 | 22.4 | 5.1 | 19.8 | 0 | 0 |
|  | Precipitation of Coldest Quarter | 0 | 0 | NA | NA | NA | NA | 0 | 0 |
|  | Precipitation of Wettest Quarter | 0 | 0 | NA | NA | NA | NA | 0 | 0 |
|  | Precipitation of Wettest Month | 2.5 | 4.9 | NA | NA | NA | NA | 4 | 2.9 |
| Measures of temperature during the driest/warmest parts of the year | Max Temperature of Warmest Month | 0.2 | 3.9 | NA | NA | NA | NA | 0.4 | 12.5 |
|  | Mean Temperature of Driest Quarter | 0 | 0 | NA | NA | NA | NA | 0 | 0 |
|  | Mean Temperature of Warmest Quarter | 0 | 0 | 0.1 | 0 | 0.9 | 1.4 | 0 | 0 |
| Measures of temperature during the wettest/coldest parts of the year | Mean Temperature of Wettest Quarter | 0.3 | 3.5 | NA | NA | NA | NA | 1.8 | 1.6 |
|  | Mean Temperature of Coldest Quarter | 0 | 0 | 1.5 | 12 | 5.6 | 12.1 | 0 | 0 |
|  | Min Temperature of Coldest Month | 0.7 | 2.3 | NA | NA | NA | NA | 0.3 | 13.4 |
| Annual Temperature | Annual Mean Temperature | 0.7 | 9.2 | NA | NA | NA | NA | 2.9 | 23.7 |
| Parent rock/soil | Rock type 1 | 11.7 | 0.9 | 11.7 | 3.5 | NA | NA | NA | NA |
|  | Rock type 2 | 7.2 | 0 | 6.6 | 0.2 | NA | NA | NA | NA |

**Appendix S5 Table 3**: Percent contribution and percent permutation of each environmental variable used to build the four different *C. concinna* MaxEnt models.  “Subset of Bioclim” indicates that only a subset of BioClim variables we used to build the model. The subset included representative variables from the correlate groups of the study area (Temperature Seasonality, Isothermality, Precipitation of the Driest Quarter, Annual Precipitation, Mean Temperature of Warmest Quarter, and Mean Temperature of Coldest Quarter). “Soil” indicates that both primary parent rock type and secondary parent rock type were used to train the model.

|  |  | Model – *Clarkia concinna* | | | | | | | |
| --- | --- | --- | --- | --- | --- | --- | --- | --- | --- |
|  |  | All Bioclim + Soil | | Minimal Bioclim + Soil | | Minimal Bioclim | | All Bioclim | |
| Bioclim Variable Correlate Group | Bioclim Variable | % Contrib | % Permut | % Contrib | % Permut | % Contrib | % Permut | % Contrib | % Permut |
| Measures of Temperature and precipitation seasonality | Temperature Seasonality | 1.8 | 3.3 | 2.2 | 1.1 | 3 | 1.5 | 3.2 | 6.2 |
|  | Temperature Annual Range | 0.5 | 0 | NA | NA | NA | NA | 0.1 | 0 |
|  | Mean Diurnal Range | 1.5 | 0.9 | NA | NA | NA | NA | 1.2 | 0.8 |
|  | Isothermality | 19.8 | 14.5 | 29.4 | 24.4 | 30.8 | 22.9 | 24 | 17 |
|  | Precipitation Seasonality | 5.9 | 12.1 | NA | NA | NA | NA | 0.4 | 17.3 |
| Measures of precipitation during the driest/warmest parts of the year | Precipitation of Driest Month | 6.1 | 9.9 | NA | NA | NA | NA | 6.8 | 7.5 |
|  | Precipitation of Driest Quarter | 5.6 | 9.7 | 19.1 | 18.6 | 17.7 | 16.3 | 9.4 | 11.3 |
|  | Precipitation of Warmest Quarter | 0.6 | 0.4 | NA | NA | NA | NA | 0.5 | 0.1 |
| Measures of precipitation during the coldest/wettest parts of the year | Annual Precipitation | 0 | 0 | 27.2 | 24.8 | 30.9 | 28 | 0 | 0.2 |
|  | Precipitation of Coldest Quarter | 0.8 | 0.2 | NA | NA | NA | NA | 0.3 | 0.2 |
|  | Precipitation of Wettest Quarter | 0 | 0 | NA | NA | NA | NA | 0 | 0 |
|  | Precipitation of Wettest Month | 38.4 | 18.4 | NA | NA | NA | NA | 39.7 | 15.7 |
| Measures of temperature during the driest/warmest parts of the year | Max Temperature of Warmest Month | 0.3 | 3.4 | NA | NA | NA | NA | 0 | 2.1 |
|  | Mean Temperature of Driest Quarter | 0.6 | 0.3 | NA | NA | NA | NA | 0 | 0 |
|  | Mean Temperature of Warmest Quarter | 0 | 0 | 0.3 | 2.9 | 0.3 | 1.7 | 1.2 | 0.3 |
| Measures of temperature during the wettest/coldest parts of the year | Mean Temperature of Wettest Quarter | 0.1 | 0.8 | NA | NA | NA | NA | 0.1 | 0.8 |
|  | Mean Temperature of Coldest Quarter | 0.8 | 3.1 | 16 | 22.5 | 17.3 | 29.6 | 0 | 0 |
|  | Min Temperature of Coldest Month | 12.2 | 17.8 | NA | NA | NA | NA | 12 | 15.5 |
| Annual Temperature | Annual Mean Temperature | 0.8 | 3.1 | NA | NA | NA | NA | 0.6 | 5 |
| Parent rock/soil | Rock type 1 | 2.4 | 1.9 | 3.5 | 3.1 | NA | NA | NA | NA |
|  | Rock type 2 | 2 | 2.8 | 2.4 | 2.6 | NA | NA | NA | NA |
