# Supplementary material for: A greenhouse experiment partially supports inferences of ecogeographic isolation from niche models of Clarkia sister species
Source: Am J Bot. 2021 Oct 18;108(10):2002–14. doi: 10.1002/ajb2.1756 (PMC9298282; doi:10.1002/ajb2.1756)
Supplement: Supplementary file 6 — Appendix S6. Maps of predictive surfaces generated by SDMs. [file AJB2-108-2002-s002.docx]

**Appendix S6:** Maps of the predictive surfaces generated by SDMs built using all 19 BioClim variables, primary rock type, and secondary rock type. Shading indicates probability of occurrence.

**
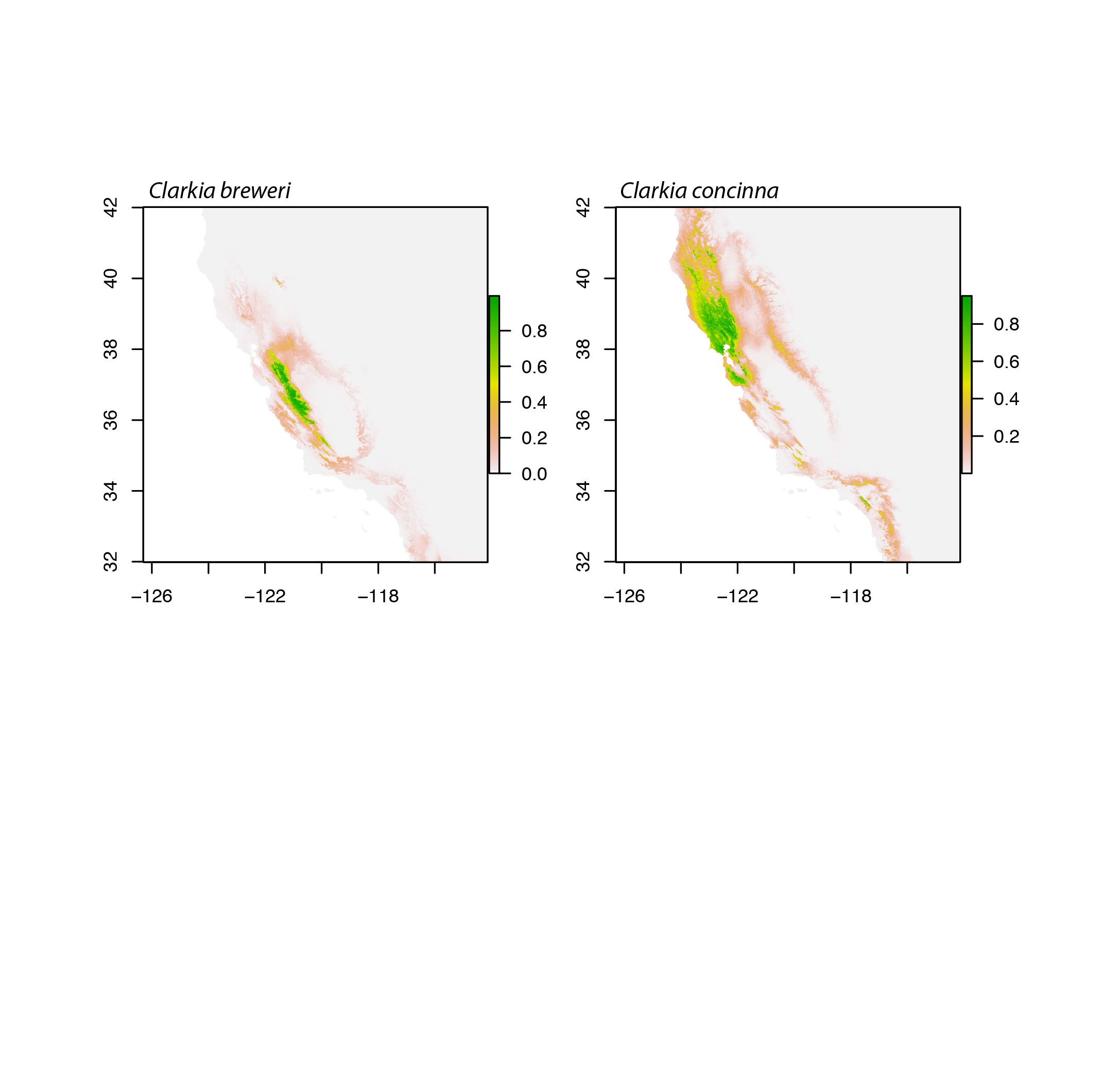
**
