# Supplementary material for: A greenhouse experiment partially supports inferences of ecogeographic isolation from niche models of Clarkia sister species
Source: Am J Bot. 2021 Oct 18;108(10):2002–14. doi: 10.1002/ajb2.1756 (PMC9298282; doi:10.1002/ajb2.1756)
Supplement: Supplementary file 7 — Appendix S7. Table of BioClim variables and PCA axis loadings. [file AJB2-108-2002-s010.docx]

**Appendix S7:** Table of Principal Components Analysis variables and loadings used to visualize axes of climate niche differentiation for *Clarkia concinna* and *Clarkia* *breweri*. BioClim variables used in the PCA are shown, along with their corresponding loading on each axis. Labels used in Figure 4 are indicated.

| **BioClim Variable:** | **Figure 4 label** | **PCA1** | **PCA2** |
| --- | --- | --- | --- |
| Annual Precipitation | Annual & winter precip | -0.1302 | -0.3055 |
| Precipitation of Coldest Quarter | Annual & winter precip | -0.0880 | -0.3226 |
| Precipitation of Wettest Month | Annual & winter precip | -0.0807 | -0.3249 |
| Precipitation of Wettest Quarter | Annual & winter precip | -0.0957 | -0.3232 |
| Annual Mean Temperature | Annual temp | 0.3218 | -0.0014 |
| Isothermality | Isothermality | 0.1124 | -0.2410 |
| Precipitation Seasonality | Precip seasonality | 0.2462 | -0.2100 |
| Precipitation of Driest Month | Summer precip | -0.3138 | 0.0541 |
| Precipitation of Driest Quarter | Summer precip | -0.3146 | -0.0214 |
| Precipitation of Warmest Quarter | Summer precip | -0.3108 | -0.0802 |
| Max Temperature of Warmest Month | Summer temp | 0.2101 | 0.2381 |
| Mean Temperature of Driest Quarter | Summer temp | 0.2741 | 0.1385 |
| Mean Temperature of Warmest Quarter | Summer temp | 0.2788 | 0.1336 |
| Mean Diurnal Range | Temp variability | -0.0118 | 0.3027 |
| Temperature Annual Range | Temp variability | -0.0744 | 0.3545 |
| Temperature Seasonality | Temp variability | -0.0839 | 0.3299 |
| Mean Temperature of Coldest Quarter | Winter temp | 0.3103 | -0.1307 |
| Mean Temperature of Wettest Quarter | Winter temp | 0.3104 | -0.1224 |
| Min Temperature of Coldest Month | Winter temp | 0.2940 | -0.1698 |
