# Supplementary material for: A greenhouse experiment partially supports inferences of ecogeographic isolation from niche models of Clarkia sister species
Source: Am J Bot. 2021 Oct 18;108(10):2002–14. doi: 10.1002/ajb2.1756 (PMC9298282; doi:10.1002/ajb2.1756)
Supplement: Supplementary file 8 — Appendix S8. Niche similarity and equivalency test graphs. [file AJB2-108-2002-s009.docx]

**Appendix S8:** Niche comparison tests using the R package ecospat. **A)** The equivalency test compares the observed niche overlap (red diamond and vertical line) to a null distribution of simulated overlaps (gray histogram) when randomly reallocating the occurrences of both species among the joint distribution of occurrences of the two species. In this case, *C. breweri* and *C. concinna* have niches that are not equivalent. **B)** The niche similarity test compares the observed niche overlap (in red) to a null distribution of simulated overlaps (in gray), given the full range of available environmental conditions represented by the pseudo-absence occurrence points. In this case, *C. breweri* and *C. concinna* are more similar than expected by chance.

**
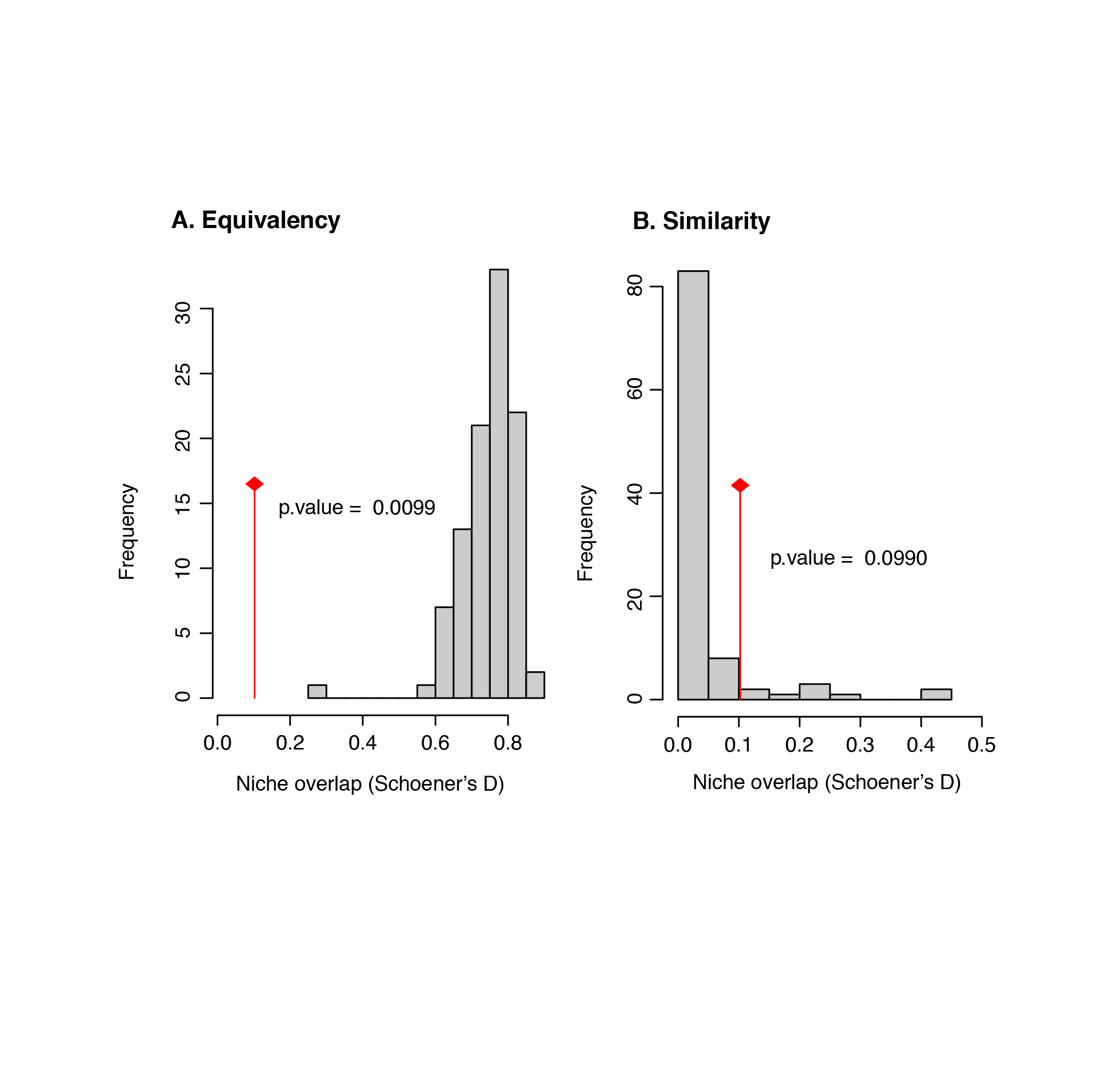
**
