# Supplementary material for: A greenhouse experiment partially supports inferences of ecogeographic isolation from niche models of Clarkia sister species
Source: Am J Bot. 2021 Oct 18;108(10):2002–14. doi: 10.1002/ajb2.1756 (PMC9298282; doi:10.1002/ajb2.1756)
Supplement: Supplementary file 9 — Appendix S9. Results tables from models used to analyze greenhouse experiment. [file AJB2-108-2002-s003.docx]

**Supplemental Appendix S9:** Models testing species or population, water treatment, soil, and interactions using number of mature seeds as a measure of lifetime fitness. Incidence rate ratio (exponentiated coefficients), 95% confidence interval and p-value shown. Significant effects (p<0.05) are shown in bold.

| **Species level model: Number of Mature Seeds (GLMM with zero-inflated negative binomial dist.)** | | | |
| --- | --- | --- | --- |
| *Predictors* | *Incidence Rate Ratios* | *95% CI* | *p* |
| **Conditional Model** |  |  |  |
| Intercept | 15.46 | 6.24 – 38.30 | **<0.001** |
| Species (*C. concinna*) | 1.56 | 0.44 – 5.56 | 0.496 |
| Water Treatment (Mesic) | 1.15 | 1.01 – 1.30 | **0.031** |
| Soil (*C. concinna*) | 1.55 | 1.23 – 1.96 | **<0.001** |
| Treatment (Mesic) x Species (*C. concinna*) Interaction | 1.37 | 1.12 – 1.67 | **0.002** |
| Soil (*C. concinna*) x Species (*C. concinna*) Interaction | 0.98 | 0.77 – 1.23 | 0.835 |
|  |  |  |  |
| **Zero-Inflated Model** |  |  |  |
| Intercept | 0.62 | 0.10 – 3.72 | 0.597 |
| Species (*C. concinna*) | 1.76 | 0.14 – 22.24 | 0.661 |
| Water Treatment (Mesic) | 0.19 | 0.09 – 0.42 | **<0.001** |
| Soil (*C. concinna*) | 1.13 | 0.51 – 2.51 | 0.756 |
| Treatment (Mesic) x Species (*C. concinna*) Interaction | 0.87 | 0.26 – 2.95 | 0.823 |
| Soil (*C. concinna*) x Species (*C. concinna*) Interaction | 0.46 | 0.13 – 1.58 | 0.218 |
|  |  |  |  |
| **Random Effects** |  |  |  |
| σ^2^ | 0.44 |  |  |
| τ_00_ _SoilSite:SoilSp_ | 0.01 |  |  |
| τ_00_ _SeedPop:SeedSp_ | 0.41 |  |  |
| ICC | 0.49 |  |  |
| N _Soil_ | 4 |  |  |
| N _SoilSp_ | 2 |  |  |
| N _SeedSource_ | 4 |  |  |
| N _SeedSp_ | 2 |  |  |
| Observations | 336 |  |  |
| Marginal R^2^ / Conditional R^2^ | 0.120 / 0.550 |  |  |

| **Population/site level model: Number of Mature Seeds (GLM with zero-infl negative binomial dist.)** | | | |
| --- | --- | --- | --- |
| *Predictors* | *Incidence Rate Ratios* | *CI* | *p* |
| **Conditional Model** |  |  |  |
| (Intercept) | 27.22 | 20.72 – 35.77 | **<0.001** |
| C2 seed source | 1.57 | 1.16 – 2.12 | **0.003** |
| B2 seed source | 0.34 | 0.23 – 0.50 | **<0.001** |
| B1 seed source | 2.11 | 1.58 – 2.82 | **<0.001** |
| Mesic treatment | 1.78 | 1.32 – 2.40 | **<0.001** |
| C2 soil | 1.04 | 0.91 – 1.18 | 0.582 |
| B2 soil | 0.77 | 0.66 – 0.90 | **0.001** |
| B1 soil | 0.58 | 0.50 – 0.67 | **<0.001** |
| C2 seed source * mesic treatment | 0.85 | 0.60 – 1.21 | 0.369 |
| B2 seed source * mesic treatment | 0.65 | 0.42 – 1.01 | 0.054 |
| B1 seed source * mesic treatment | 0.65 | 0.47 – 0.90 | **0.009** |
|  |  |  |  |
| **Zero-Inflated Model** |  |  |  |
| (Intercept) | 1.94 | 0.78 – 4.80 | 0.154 |
| C2 seed source | 0.13 | 0.04 – 0.43 | **0.001** |
| B2 seed source | 1.59 | 0.53 – 4.84 | 0.409 |
| B1 seed source | 0.05 | 0.01 – 0.17 | **<0.001** |
| Mesic treatment | 0.19 | 0.06 – 0.61 | **0.005** |
| C2 soil | 1.13 | 0.48 – 2.67 | 0.785 |
| B2 soil | 0.90 | 0.42 – 1.94 | 0.789 |
| B1 soil | 2.63 | 0.99 – 6.96 | 0.052 |
| C2 seed source * mesic treatment | 0.76 | 0.10 – 5.64 | 0.790 |
| B2 seed source * mesic treatment | 0.74 | 0.17 – 3.24 | 0.685 |
| B1 seed source * mesic treatment | 2.04 | 0.33 – 12.69 | 0.445 |
| Observations | 336 |  |  |
| R^2^ / R^2^ adjusted | 0.998 / 0.998 |  |  |
