# Supplementary material for: A greenhouse experiment partially supports inferences of ecogeographic isolation from niche models of Clarkia sister species
Source: Am J Bot. 2021 Oct 18;108(10):2002–14. doi: 10.1002/ajb2.1756 (PMC9298282; doi:10.1002/ajb2.1756)
Supplement: Supplementary file 10 — Appendix S10. Soil chemistry and water‐holding capacity table. [file AJB2-108-2002-s007.docx]

**Supplemental Appendix S10:** Results of soil chemistry analysis and water holding capacity from *Clarkia* *concinna* populations Chiles Valley (C1) and Knoxville Road (C2), and *Clarkia breweri* populations Mount Hamilton (B1) and Mile Marker 8 (B2).

|  | Chiles Valley **(C1)** | Knoxville Road **(C2)** | Mount Hamilton **(B1)** | Mile Marker 8 **(B2)** |
| --- | --- | --- | --- | --- |
| % Organic Matter Rating | 3.2 | 2.5 | 1.2 | 3.9 |
| Estimated N release (lbs/A) | 93 | 80 | 53 | 108 |
| P (ppm) | 6 | 8 | 9 | 79 |
| NaHCO3P (ppm) | 19 | 7 | 7 | 19 |
| K (ppm) | 87 | 2 | 86 | 92 |
| Mg (ppm) | 1012 | 7 | 393 | 107 |
| Ca (ppm) | 333 | 1003 | 948 | 1050 |
| Ca/Mg Ratio | 0.33 | 143.29 | 2.41 | 9.81 |
| Na (ppm) | 14 | 4 | 31 | 12 |
| Soil pH | 7 | 6.5 | 6.1 | 5.6 |
| H (meq/100g) | 0 | 0.4 | 1.4 | 2 |
| Cation exchange capacity (meq/100g) | 10.3 | 5.5 | 9.7 | 8.4 |
| % K saturation | 2.2 | 0.1 | 2.3 | 2.8 |
| % Mg saturation | 81 | 1.1 | 33.4 | 10.5 |
| % Ca saturation | 16.2 | 91 | 48.9 | 62.5 |
| % H saturation | 0 | 7.5 | 14 | 23.5 |
| % Na saturation | 0.6 | 0.3 | 1.4 | 0.6 |
| NO3N (ppm) | 14 | 22 | 3 | 9 |
| SO4S (ppm) | 6 | 6 | 14 | 2 |
| Zn (ppm) | 1.8 | 1 | 3.4 | 0.5 |
| Mn (ppm) | 4 | 5 | 8 | 12 |
| Fe (ppm) | 18 | 16 | 29 | 13 |
| Cu (ppm) | 0.7 | 1.6 | 0.8 | 0.3 |
| B (ppm) | 0.5 | 0.4 | 0.4 | 0.3 |
| Soluble salts (mmhos/cm) | 0.3 | 0.5 | 0.3 | 0.2 |
| Mean percent water holding capacity | 42.64 | 10.39 | 6.11 | 15.40 |
